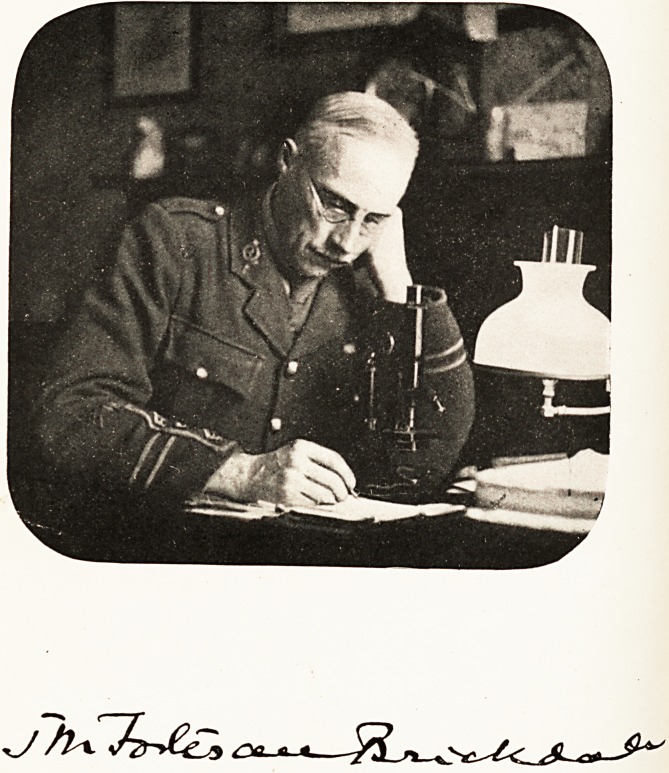# John Matthew Fortescue-Brickdale

**Published:** 1921-06

**Authors:** 


J M Fortescue Brickdale

### Obituary.

### JOHN MATTHEW FORTESCUE-BRICKDALE, M.A., M.D. Oxon., M.R.C.P. Lond.

It is with profound regret that we record the death of our Editorial Secretary, Dr. Fortescue-Brickdale, on June 2nd, at the early age of 51, after an illness whose final stages were unexpectedly short. For although it was clear to his friends that his health had been seriously impaired by severe illness whilst serving in France, it was with almost dramatic suddenness that the disease assumed a new and, as it proved, fatal aspect. Brickdale, who had been educated at Dulwich, Guy's and Oxford, came to Bristol in 1903, after leaving Uppingham, where he had been school doctor. His scientific knowledge and personal charm soon brought him into public affairs. His first medical election here was to the staff of the Children's Hospital: then in fairly rapid succession he was appointed Lecturer in Pharmacology in the University of Oxford, Assistant Physician to the Bristol Royal Infirmary, Director of the Public Health Laboratory in the University of Bristol, Physician to Clifton College, and finally, in 1919, Physician to the Royal Infirmary. There had been an interesting connection between his family and the Royal Infirmary: " In 1766 Matthew Brickdale wrote to the Committee stating that his uncle, John Brickdale, had left £200 to the Infirmary on condition that his great-nephew (the said Matthew) and his heirs should have the ' Power and Privelage of perpetual subscribers.' This condition was agreed to. In 1840 John Fortescue-Brickdale successfully claimed this right as a descendant of Matthew Brickdale. In 1867 Matthew Inglett Fortescue-Brickdale's name was substituted for that of his late father in the list of life trustees."

Dr. Brickdale was the younger son of the last named, and his loyal affection for the old B.R.I. was perhaps a greater asset to the place than the " Power and Privelage " to which he was in some sense the heir. During the war Brickdale served as a captain in the R.A.M.C.(T.), first at the 2nd Southern General Hospital in Bristol and later for about two years in France, where his health became so seriously undermined. On his return to Bristol he was given charge of a special centre at Southmead, where he studied the later phases of "Chest Wounds," publishing an admirable summary of his observations in the *Quarterly Journal of Medicine* in 1918. Brickdale had wide sympathies and many interests. He was a keen member of the Bristol Medical Book Club, whose antiquity appealed strongly to his tastes. He was a zealous churchman and at the time of his death was Churchwarden at All Saints', Clifton. On occasion he had filled a part in the Medical Dramatic performances, his real talent in this line being somewhat overshadowed by memories of the terrific success which attended his delivery of the simple phrase, " The prisoner refuses to answer."

All his colleagues as well as his many friends will miss sadly his genial wit, his sallies of erudition, and above all his sweet reasonableness. We are indebted to a friend and contemporary of his at Oxford for the following personal tribute to his memory :—

" Jack " Fortescue-Brickdale at Oxford, where I first knew him (we went up to Christ Church in the same term, October, 1888), was a lovable figure. How well I remember going to his rooms, in Meadow Buildings, for the first time. Our talk was of literature and life (little enough we both knew of it then), and it was to him that I owed my introduction to Lamb's inimitable prose. I think I see that afternoon now : Jack bustling about getting tea, after having handed me the *Essays of Elia* and having pointed out some exquisite passage from " Oxford in the Vacation," or some daring witticism in " Distant Correspondents," an essay in which he specially delighted. I can truthfully say that, although I had many friends at the House even in those early days, it was Jack's engaging personality that chiefly attracted me, and my memories of Oxford are so indissolubly wrapped up with him that it is impossible for me to separate him from the Home of Lost Causes. We became inseparable ; by casual acquaintances we were constantly mistaken for each other, much to my advantage in every way, and how greatly I benefited by his dear comradeship it is impossible for me to say.

Jack was one of those men whose minds are so alert, that if one uttered some paradox or gave vent to some ridiculous association of ideas, he would catch it up and carry on the pleasant fiction in the gayest and most delightful way. He was never one to reply monosyllabically to any remark ; he had something of the touchstone in his composition ; full of wit himself, he was as often the cause of wit in others. How we have sat, with kindred spirits, a hundred times into the early hours of the morning and, to use Henley's phrase, have mapped " the course of man's regeneration over a pipe!" How often have I not returned to my rooms in Canterbury, with the moon lighting up the delicate tracery of Wren's tower, full of the immediate memory of those "noctes." How one regretted that such symposia were not eternal; even the imminent "vac" lost something of its glamour in the greater glamour of that delightful and frequent companionship. In music we had another bond. Jack played the violin better than the average amateur, and his knowledge of the great masters of tone was equal to his acquaintance with the great masters of poetry and prose. The Musical Union under old Johnny Mee, as the Rev. John Mee, D.D., Precentor of Chichester, etc., etc., was familiarly known to us, was then in the hey-day of its success, and Jack was one of those who had a standing invitation to the Sunday meetings at Kettel Hall which the Doctor extended to a few favoured ones. We liked those re-unions, not only because of the concord of sweet sounds and the host's genial welcome, but because it gave us House men a certain *kudos*—we had some twenty minutes (was it? or was it longer?) more grace than members of other colleges, and we rather ostentatiously stayed out our welcome, (I trust we did not over-stay it) in order to show, I fear, our superiority, or rather the superiority in sense possessed by those who framed the laws of Christ Church. Jack loved this—and, after all, we were but boys. He left Oxford, as he had entered it, trailing clouds of glory. In the larger world I did not, at first, see so much of him; but he told me, wondering, that he was going in for medicine; and lo! at Guy's he was the most brilliant man of his year. Of course he was. I was in no way surprised. After a short period at Uppingham he settled in Bristol, and I saw still less of him; but whenever we did meet he was always the same dear fellow, amusing and delightful; and when he wrote, his letters still contained the same boyish jokes and banter. Work and worry may have aged him in outward appearance; the war, in which he fought against disease and death as nobly as did any combatant against the other enemies, left its mark upon him, but he died as he had lived a boy at heart:—

> " Seasons impaired not the ray
> Of his buoyant cheerfulness clear."

In spite of all the ills that flesh is heir to, in spite of one of the heaviest blows a man can be called upon to encounter, Jack died young, and he carried to his grave on the hill that looks towards the Cotswolds and the Mendips, where to-morrow's rising sun will gild his new-made grave, the love of those who knew him best, and who as they stood around his last sleep, could say with his favourite poet :—

"How he lies in his rights of a man,
Death has done all Death can."

### Local Medical Notes.

## Figures and Tables

**Figure f1:**